# Physical stress triggers in simulated emergency care situations

**DOI:** 10.1002/nop2.614

**Published:** 2020-08-31

**Authors:** Anna Abelsson, Marcus Gustafsson, Christina Petersèn, Susanne Knutsson

**Affiliations:** ^1^ Department of Nursing Science School of Health Sciences Jönköping University Jönköping Sweden; ^2^ Department of Health and Caring Sciences Faculty of Health and Life Sciences Linnaeus University Växjö Sweden

**Keywords:** education, electrodermal activity, galvanic skin response, intervention, nurse, simulation

## Abstract

**Aim:**

To practise emergency care situations during the education can be stressful. The aim of this study is to identify factors that cause stress in simulated emergency care.

**Design:**

A descriptive observational study.

**Methods:**

Video recordings (*N* = 26) subjected to observation with written field notes in turn subjected to interpretive qualitative content analysis.

**Results:**

To assess the patient's condition and decide what measures to take trigger stress reactions. If the students failed to connect the correct and relevant information in the conversation with the physician, the students showed signs of stress. Also, to calculate medication dosages stress the students.

## 1 INTRODUCTION

A nurse's knowledge, skills and experience are of great importance for ensuring good and safe care. Thus, it is important that nurses in training are given the opportunity to practise, for example emergency care situations during their education (Abelsson, Rystedt, Suserud, & Lindwall, 2018). Practice to become a clinically competent nurse in educational settings and the assessment of competence is therefore important as it ensures that the individual knows what is required of them when carrying out their profession (Miller, 1990). Marton and Booth (1997) believe that knowledge is to know and to understand something. Aristotle (2000) also describes knowledge as a skill used in practical action. Gadamer (1989) describes how knowledge leads to further knowledge and experiences. With pre‐knowledge and familiarity within an area, understanding and interpretation can lead to new knowledge beyond our existing meaning horizon. Knowledge and experience are created in a circle where the interpreter of a phenomenon creates a design of meaning by moving from the whole (the theorem) to the part (the set pieces) and back to the whole (Gadamer, 1989).

## 2 BACKGROUND

With simulation, theoretical and practical knowledge can be trained to become experiences. Simulation is, therefore, increasingly used in nurse education. In situ simulations are common as they include the demonstration of skills and show the participants' performance of an independent clinical practitioner, according to Miller's competence pyramid (1990). Simulation, however, exposes students to different degrees of stress and anxiety (Abelsson & Bisholt, 2017; Abelsson et al., 2018). The complexity of the simulated environment determines the level of stress experienced by the participants. At a medically advanced level, the more complex environment with many simultaneous events, the more complicated the simulation and thus increasing levels of stress (Abelsson et al., 2018). If the simulated scenarios have relevant content and are not exaggerated, participants maintain a comfortable stress level (Abelsson & Bisholt, 2017). By minimizing the nursing students' stress, the learning improves (Miller & Sawatzky, 2017).

Experiencing stress is not necessarily a negative condition. A moderate sympathikus surcharge helps the person perform over their normal cognitive ability (Gradari, Pallé, McGreevy, Fontán‐Lozano, & Trejo, 2016). It can also improve the possibility of learning for the individual (Al Sabei & Lasater, 2016). When exposed to stress, the sympathetic nervous system is activated in response to the demands made against the personal resources available. If the requirements outweigh the resources needed, the person will experience stress through a sympathikus surcharge (Timmermans, Xiong, Hoogenraad, & Krugers, 2013). However, if there is too much stress, it will result in a worsening cognitive capacity of the individual (Merz, Dietsch, & Schneider, 2016; Nourbakhsh, Chen, Wang, & Calvo, 2017). Unknown factors in the clinical environment can cause anxiety and produce negative effects on nursing students’ cognitive ability, such as concentration and problem‐solving (Baksi, Gumus, & Zengin, 2017). A cognitive overload during complex and demanding tasks leads instead to impaired performance (Merz et al., 2016; Nourbakhsh et al., 2017).

In this study, the authors wanted to identify how to enhance learning experience during the simulation and, as a first step, sought to identify factors that cause stress. The aim of this study is to identify factors that cause stress in simulated emergency care.

## 3 METHODS

### 3.1 Design

This study was designed as a qualitative, descriptive and observational study with readings of individual electrodermal activity and the fluctuations correlated with specific events during a simulation of emergency care in a hospital ward. The students were observed in the simulation situation using video recordings, focusing on how students acted and interacted with the patient, other team members and the environment. This gave an understanding of what was happening when physical stress was triggered, and the students’ actions were put in context. The reporting follows the guidelines according to Cheng et al. (2016).

### 3.2 Participants

The participants in this study consisted of nursing students at a university in southern Sweden. The inclusion criteria were nursing students participating in a simulation in the sixth semester of the nursing programme. In total, 40 students were invited to participate and 26 consented; 20 women and six men, with a mean age of 30 years. The students had no previous experience of being responsible for the emergency care of a patient in clinical settings, as during their clinical training, a supervisory nurse was always present. During simulated care at the university, only a few emergency care scenarios have been completed and the students, therefore, have limited experience of simulating scenarios with emergency care situations. The participants were in no way dependent on the researcher and were informed of the aim of the research.

### 3.3 Setting

The simulations were performed at the clinical training facility at the university. The scenarios focused on an emergency care situation. An inpatient at the hospital ward, emitted for angina, experienced sudden chest pain. The patient rang the bell, and the students answered the call. The students were required to examine the patient and to identify the reason for the chest pains. They were then required to call the physician for the prescription of medicine. Each scenario was performed by two students, one student as the primary nurse with medical responsibility for the patient's care and the second student as a colleague to help out in the situation. The physician's phone was answered by the responsible teacher at the clinical training facility, familiar with each scenario. The patient was a high‐fidelity, talking manikin, manoeuvred from an adjacent room. The researchers took the role of observers from the same adjacent room. The 26 scenarios lasted for 18–35 min (median = 23 min). The debriefing was not included in the data material.

### 3.4 Data collection

#### 3.4.1 Video recording

Three video cameras were used to record three different viewpoints and offer more opportunities to collect information, both verbal and non‐verbal communication that occurred during each simulation. One camera was fixed at the primary nurse student's forehead, another at the top of the patient's bed, and a third camera was placed on a tripod in the ceiling corner of the room. The placement of all three cameras was carefully thought out to reduce the risk of disturbing the students.

#### 3.4.2 Empatica Wristband

The primary nurse student wore an Empatica E4 Wristband during the scenario. The Empatica E4 Wristband measures the following: heart rate, peripheral skin temperature, acceleration, which is the body and arm movements and electrodermal activity. The autonomic nervous system activities are registered through a person's electrodermal activity. The registration of increased humidity, initiated at an intuitive level by the sympathetic nervous system, alters the skin's conductivity which is depending on attentional, emotional and motivational processes in the nervous system that takes place during emotional arousal (Phitayakorn, Minehart, Pian‐Smith, Hemingway, & Petrusa, 2015). The registration of the autonomic nervous system activity shows the person's cognitive load in real time (Nourbakhshet al., 2017). An increase of the cognitive load triggers a stress reaction shown as a rapidly ascending and descending curve (Benedek, & Kaernbach, 2010).

#### 3.4.3 Data analysis

In this study, the video recordings in relation to the students’ electrodermal activity were first subjected to observation with written field notes. The field notes were then subjected to content analysis.

#### 3.4.4 Video observation

In the study, field notes were made during observation of the video recordings in relation to the registered autonomic nervous system activity shown as a rapidly ascending curve of the electrodermal activity that then descends. During the observations, the focus was on the actions the students were taking part in when the electrodermal activity curve ascended, representing a stressful situation. The field notes included the students’ reactions and interactions in the different situations where the electrodermal activity curve ascended. The notes included what was said in the room (Emerson, Fretz, & Shaw, 2011). The researcher's reflections during the observations and questions to follow up during the next observation were also noted (Abelsson & Bisholt, 2017). Three researchers, all with prior experience of simulation as an educational method, were the primary assessor of eight recordings and with the role of the secondary assessor in the remaining 16 recordings.

#### 3.4.5 Content analysis

An interpretive qualitative content analysis was used (Krippendorff, 2012). The analysis started with familiarizing of the transcribed data by reading with an open mind, to reach an understanding of the substance of the data. The text was then read carefully to identify meaning units that represented the aim of the research. Codes were then derived from the meaning units. The codes were then abstracted to subcategories based on similarities and differences and sorted into categories. The relevance of the results was finally verified by the correlation between the aim of the research and the categories (Krippendorff, 2012).

## 4 RESULTS

The researchers identified what triggered physical stress reactions for nursing students in simulated emergency care of a patient. The result is presented in two categories:*When responsibility becomes a reality* and *When knowledge does not correspond to demands*. Selected quotes from the written field notes are presented in the results.

### 4.1 When responsibility becomes reality

When the patient rang a bell in the room, a stress reaction was triggered in the students. The students now took over responsibility for the patient and the situation. The situation became serious, and the students had a stress reaction when taking responsibility for the patient's care and health:

The student starts talking to the patient somewhat cautiously. Now, the student starts to realize the seriousness of the situation. The responsibility for assessment and care now falls on the student.

In the patient's room, another stress reaction was triggered when the students started checking the patient's identity. The student was responsible for verifying the patient's identity and confirming that they were treating the right patient:

The student lifts the patient's arm and looks at the identity bracelet while also asking the patient for his date of birth.

The students delegated caring tasks to their fellow students, which triggered a physical stress reaction. The delegation could be to connect the patient to the monitoring devices, fetching an ECG machine, or adjusting the bed to a more comfortable position for the patient:

The student asks fellow students if they would like to read the vital signs. The question is not interpreted as a delegation, so the fellow students first busy themselves with the patient and then read vital signs.

### 4.2 When knowledge does not correspond to demands

When the students evaluated and re‐evaluated the patient, gathering vital parameters from the monitoring equipment or from fellow students, a stress reaction was triggered. The patient's condition was deteriorating according to the vital parameters, and the care measures taken had not been enough:

The patient says his chest pains are growing worse. He has trouble breathing. The student now sees that the patient has a cold sweat and breathes heavily. The student realizes that the patient is deteriorating, but she doesn’t know what to do.

When the patient showed anxiety and fear, a stress reaction was triggered for the students. During the stress reaction, the students could ask fellow students to calm down a patient who was in fact dozing:

He is very anxious, the student repeatedly says while the patient lies quietly in bed.

When students made a decision about continued treatment, for example more Nitrolingual, or when they used their fellow students in the conversation about and planning for continued treatment, repeated stress reactions were triggered. The situation required knowledge to handle the increasingly acute care situation. The patient's condition deteriorated, and the student requested oral help handling the situation:

The patient expresses that he is in more pain. The student realizes that the patient is ill, but he doesn't know what to do. ‘I’m sure the physician will be here soon, the student says while pacing the room.

In situations where students asked fellow students whether they still had contact with the patient, whether he was conscious, a stress reaction was triggered. The patient's general condition deteriorated, and the patient was about to die, which triggered stress reactions.

Communication with the physician resulted in triggered stress response in students. Both the decision on making the call and the call itself caused stress reactions. During the call, the students did not connect correct and relevant information in the conversation which triggered stress reactions:

The student calls the physician.

The student sighs loudly while waiting for the physician to pick up.

When the telephone call to the physician did not work, stress reactions were triggered in the students. When the physician's phone was busy, stress reactions were triggered. Some students called back immediately, while others chose to read patient notes or go back to the patient room. No matter the strategy, stress reactions were triggered by not receiving a reply from the physician:

The physician’s phone is busy. The student calls back twice more, but it’s still busy. The student sits by the desk and starts scribbling intensely in her notebook. She doesn’t know what to do in this situation.

When the physician answered the phone, a stress reaction was also triggered. The students did not always report essential information to the physician, nor did they document the physician's prescription. Regardless of the outcome of the call with the physician, the student had a stress reaction:

The student reports to the physician. She is unsure with what she's saying and there’s uncertainty in her voice. She gives no direct information about the patient to the physician.

Each time the physician asked follow‐up questions, a stress reaction was triggered:

‘The patient’s condition is worsening,’ the student tells the physician over the phone. ‘What are the patient’s vital parameters?’ the physician asks. The student becomes nervous and fidgets in his chair. He can’t answer the question.

In the medication room, stress reactions were triggered repeatedly. Looking in the medication cabinet took time and was done without systematic considerations, such as alphabetical order or placement of class A drugs. The students lacked the ability to handle medication or calculate the correct dosage. The students did not reflect out loud on what medication was to be given or in what dosage. Stress reactions were triggered for the students when the patients became progressively worse and the medication had to be prepared in a short amount of time:

The student is looking in the medication cabinet. She can't find what she's looking for and says aloud: ‘I hope he's alive then'.

A stress reaction was triggered when the students pottered aimlessly with the needles, picked up and put away different syringes and needles several times:

The student has her needle and her medication vial in one hand. She continues looking aimlessly about in the medication cabinet.

When the students calculate the correct medication dosage and double‐checked their medication vial on several occasions, repeated stress reactions were triggered. Another stress reaction was triggered when the students mumbled aloud to themselves to confirm their thoughts. The students repeated medication names, medication potency, the prescribed dose and ways to administer the medication:

The student takes out the medication and keeps talking aloud to herself to confirm what she's doing.

The students hesitated about ways to administer medication, whether it was to be given intravenously or subcutaneously which triggered a stress reaction. Not having access to the physician's written, documented prescriptions, nor having any notes of their own after the call with the physician, triggered a stress reaction:

The student doesn’t know what size of the needle she should use, because she missed taking a note on whether the prescription was to administer intra‐muscularly or subcutaneously.

Both when the medication was documented in the Class A drug chart and when the students double‐checked the medication dosage by calculating it before it was administered to the patient, a stress reaction was triggered:

The student looks at the syringe, writes intensively in the Class A drug chart, looks at the syringe, writes intensely again. She focuses on looking at the syringe while she processes how much medication she has used compared to what the prescription said.

On the occasions that the students realized that the medication dosage in the syringe was wrong, another stress reaction was triggered. Another stress reaction was triggered if the patient commented that he or she was feeling worse after receiving medication, for example experiencing nausea or light‐headedness:

When the student has administered the morphine, the patient says that he is dizzy and feels a bit nauseous. The student asks him about his heart medication and takes another blood pressure.

A stress reaction was triggered when the students looked for other causes than the effect and side effects of the medications, for the patient's sudden deteriorating vital parameters. A stress reaction was triggered when the students expressed that they lacked sufficient knowledge and did not have control over the situation:

The student says aloud: 'I don't know what to do.’

The students who did know the effects of the medication also showed stress reactions when the patient reacted to the medication through, for example, a drop in blood pressure.

## 5 DISCUSSION

According to Gadamer's knowledge circle (1989), pre‐knowledge and familiarity within the area, understanding and interpretation generate new knowledge beyond the students existing meaning horizon. When the learning circle can become a gradual process over time, the nurse student can gradually gain experience (Mellor & Gregoric, 2016). Therefore, the students need to go through skill practice and skill evaluation before they are given more advanced scenarios to simulate. By introducing simulation in the first semester in the education programme, the students could become used to simulation, something Nielsen and Harder (2013) claim moderate anxiety and improve learning. The simulation at the beginning of the nurse education should focus on non‐technical skills. The technical skills could be focused on later in education when the students have recorded appropriate theoretical knowledge and practical skills (Nielsen & Harder, 2013).

The involvement of practicing clinical nurses in the simulations could link the students with the realities of nursing practice (Liaw et al., 2014). This may be needed to prepare them for the role of clinical practice. Because when the patient rang a bell, the responsibility of being a nurse became a reality for the nurse students. This may be one of the first times they became aware of the seriousness of the situation, of being responsible for the care of a patient. Because it is during nurse education, the student's clinical confidence and a consolidation of their clinical skills shall have been built (Blevins, 2018). The findings in this study show how students have stress reactions when managing the complexities of the patient care. Students show stress when applying their knowledge and skills to the medical, psychological and social problems (Ter Maten‐Speksnijder, Grypdonck, Pool, Meurs, & Van Staa, 2015).

To cope with the situation, the amount of responsibility in the scenario needs to be suitable for the students to handle (Kaihlanen, Haavisto, Strandell‐Laine, & Salminen, 2018). When the students have a positive feeling and self‐confidence, their cognitive ability to process information during simulation increases. This means that they will perform better in the scenario. The students need to know what is expected of them in a safe academic environment with supportive teachers (Janzen et al., 2016). But as pointed out by Nash and Harvey (2017), the transfer of simulation learning to practise cannot be assumed, due to the clinical placements are in settings that are dissimilar to the simulation scenarios.

The present study showed that the students had stress reactions when using skills in different areas. When students are to perform care acts that they do not have adequate training in, their cognitive ability is impaired and they do not function at a normal level during the scenario (Sarason, Pierce, & Sarason, 1996). Most likely, the students had increased stress levels already before the start of the simulation due to the prospect of needing to be in the spotlight (Abelsson, 2019). This triggered stress reaction impairs their ability to process information and at the same time learn in the situation (Al Sabei & Lasater, 2016). By becoming more used to simulating, the participants can act with more confidence, which can also enable learning (Abelsson, 2019).

The results show that having little training in delegating tasks to fellow students triggered stress reactions. It is important that the students learn to delegate tasks since delegation is a part of the nurse's scope of practice. The delegation means that the nurse is accountable and responsible for the nursing practice (ANA, 2012). The students in the study know that the responsibility for a delegation is based on their judgment. It creates uncertainty because the students lack the knowledge to handle the complexity of the patient's care. Nor can they determine which type and intensity of supervision are required by the fellow students or of the patient (ANA, 2012; Ulrich, 2014). The responsibility of delegation also includes learning to communicate with colleagues and patients independently, which nursing students may need to develop (Kaihlanen et al., 2018).

### 5.1 Limitations

The students were already physiologically activated before the scenario began, which Phitayakorn et al. (2015) claimed is due to anxiety regarding the upcoming simulation. In the present study, the baseline was, therefore, not measured before, as it would have required an extended rest before the simulation. This can be considered a limitation in the study.

### 5.2 Implications

To learn how to manage different situations in a hospital ward, the students are helped by practice in a clinical setting. Simulation of care situations that become more acute is a way for nurse students to practise and experience deteriorating patients. With experience, the stress reaction can be handled by the students. Repeated exposure to scenarios during nurse education makes students familiar with the scenarios, which may reduce stress reactions and enhance learning. If the scenarios are adapted to the students' theoretical knowledge and skill level, the stress reactions may be manageable. When the conditions for simulation are made apparent in advance and all disruptive or irrelevant events in the scenarios are avoided, the learning situation may enhance for the students. The scenarios should focus on factors that the students need to learn and irrelevant factors are minimized. A well‐adapted scenario may decrease the trigger situations and make learning more accessible for students in simulated care situations.

## 6 CONCLUSION

To avoid physical stress reactions for students in simulated emergency care, repeated exposure to scenarios, adapted to the students’ theoretical knowledge and skill level, should be set up. This creates simulation skills among the students, reduces stress reactions and thereby enhances learning. A well‐adapted cognitive load can better enable learning for students in simulated emergency situations.

## CONFLICT OF INTEREST

The authors declare that they have no conflicts of interest to report. All authors have read and approved the final version.

## ETHICAL APPROVAL

The study followed the ethical principles according to the World Medical Association (2013) about anonymity, integrity and maintaining public confidence. Ethical approval was not needed according to Swedish law (SFS, 2008008:192). Informed consent was obtained from each participant.

## Data Availability

All data analysed during this study are included in this published article.
